# Unsupervised IMU-based evaluation of at-home exercise programmes: a feasibility study

**DOI:** 10.1186/s13102-022-00417-1

**Published:** 2022-02-19

**Authors:** Dimitrios-Sokratis Komaris, Georgia Tarfali, Brendan O’Flynn, Salvatore Tedesco

**Affiliations:** 1 Tyndall National Institute, University College Cork, Lee Maltings Complex Dyke Parade, Cork, T12 R5CP Ireland; 2 School of Health Sciences, Queen Margaret University, Edinburgh, Scotland

**Keywords:** Performance assessment, Accelerometer, Movement quality, Exercise adherence

## Abstract

**Background:**

The benefits to be obtained from home-based physical therapy programmes are dependent on the proper execution of physiotherapy exercises during unsupervised treatment. Wearable sensors and appropriate movement-related metrics may be used to determine at-home exercise performance and compliance to a physical therapy program.

**Methods:**

A total of thirty healthy volunteers (mean age of 31 years) had their movements captured using wearable inertial measurement units (IMUs), after video recordings of five different exercises with varying levels of complexity were demonstrated to them. Participants were then given wearable sensors to enable a second unsupervised data capture at home. Movement performance between the participants’ recordings was assessed with metrics of movement smoothness, intensity, consistency and control.

**Results:**

In general, subjects executed all exercises similarly when recording at home and as compared with their performance in the lab. However, participants executed all movements faster compared to the physiotherapist’s demonstrations, indicating the need of a wearable system with user feedback that will set the pace of movement.

**Conclusion:**

In light of the Covid-19 pandemic and the imperative transition towards remote consultation and tele-rehabilitation, this work aims to promote new tools and methods for the assessment of adherence to home-based physical therapy programmes. The studied IMU-derived features have shown adequate sensitivity to evaluate home-based programmes in an unsupervised manner. Cost-effective wearables, such as the one presented in this study, can support therapeutic exercises that ought to be performed with appropriate speed, intensity, smoothness and range of motion.

## Introduction

Recovery from surgical operations, trauma or musculoskeletal disorders is heavily reliant on patient involvement in a physical therapy programme which is often extended to home-based settings. What is more, the global COVID-19 pandemic has caused sweeping disruptions to all aspects of global health systems and propelled the transition towards remote, accessible consultation and home-based rehabilitation [1]. Tracking home exercise compliance and performance quality are two critical aspects ensuring an effective rehabilitation program. Home-based exercise performance has been routinely evaluated either retrospectively through patients’ self-reports, diaries and log-books [2–7], or by assessor ratings of participants performing in video recordings [8, 9] or live sessions [10, 11]. However, such assessment methods and tools are also likely to be severely biased and inaccurate [12]. For example, patients tend to over-report the number of times they carried out exercises when at-home [5, 13], which underlines the need of an unbiased and objective method for the monitoring and assessment of effectiveness of home rehabilitation programmes. Furthermore, studies with multiple assessors with dissimilar expertise or with specialists rating live performance, may report findings that are prone to experimental bias, particularly when the investigators are not blinded to group allocation [8].

In contrast to the conventional assessment of patient performance by the appointed physiotherapist, wearable technologies and easy-to-understand metrics can enable the automated, unsupervised and objective evaluation of home-based exercise programmes, along with the patients’ compliance with the prescribed treatment plan. Emerging wearable technologies designed for movement monitoring and tele-rehabilitation typically consist of inertial measurement units (IMUs), step activity monitors [14], electromyography (EMG) and electrical muscle stimulation (EMS) sensors [15], and can operate in conjunction with virtual reality systems [16] and mobile phone applications [15]. With regards to the features and algorithms that accompany such systems, authors have previously relied on metrics measuring the duration of each exercise session, the number of the correctly performed repetitions of an exercise [14, 17], and the exercise performance quality (e.g., a measure of “distance” of the data recorded by the patient from a specific baseline expressed in terms of root-mean square distance, norm of jerk or log-likelihood [18]).

In view of the methodological limitations of previous works assessing compliance and performance quality during unsupervised at-home rehabilitation, the purpose of this feasibility study was to determine whether exercise performance at home for a physical therapy program can be determined by the use of an IMU sensor. For the evaluation of therapy motions, we tested multiple IMU-derived features assessing movement duration, smoothness, intensity, regularity, and control. The developed wearable system and the accompanying algorithms were deployed during a week-long programme with healthy participants. Similar to Allen, Bongiorni [19] and Bade and Stevens-Lapsley [20], we opted for strengthening and cardiorespiratory endurance exercises, targeting the main muscle groups of the lower limbs and core, tailored to musculoskeletal disorders and performed in a safe and controlled manner.

## Material and methods

### Participants and data collection

Thirty healthy volunteers were recruited from the university’s staff, community groups and social clubs (20 males and 10 females; mass: 72.0 ± 12.8 kg; height: 167.2 ± 32.4 cm; age: 31.0 ± 3.7 years). Participants (one volunteer) were excluded if they reported any previous musculoskeletal disorder, pain or discomfort. Detailed anonymised anthropometric measurements (dominant leg, weight, height, age and sex), data collection dates and group allocation are also included in the public repository files associated with this study. The study had the ethical approval of the university’s ethics committee (CREC reference number: ECM 4 13/08/19) and all participants gave written informed consent.

**Fig. 1:**
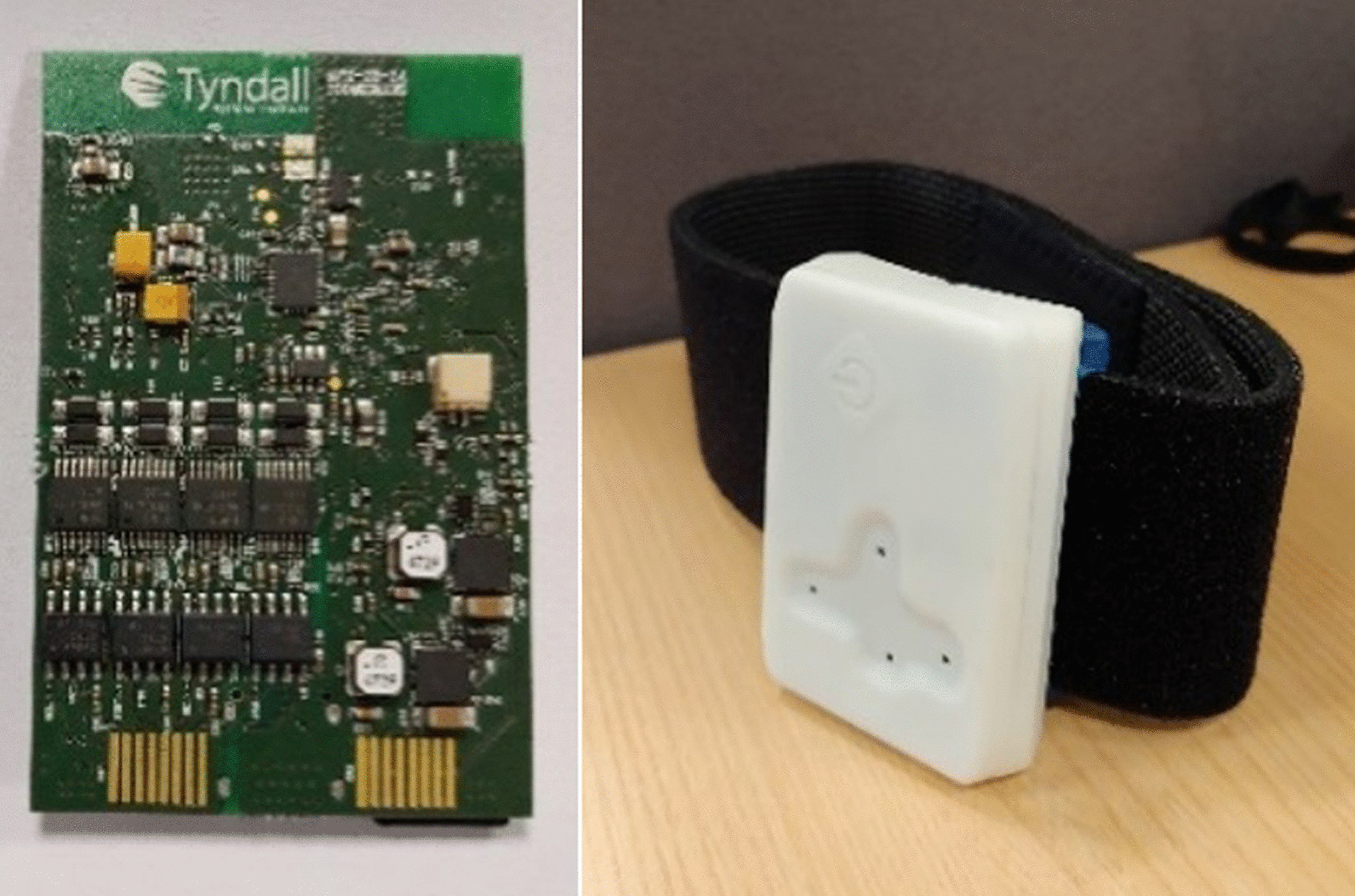
PCB board (left) and 3D printed enclosed device with Velcro strap (right)

Participants were asked to attend a single lab session and perform typical physiotherapy exercises. Movements were captured with a bespoke sensor that was attached on the participants’ dominant leg (self-reported, i.e., the leg used to kick a football) with a Velcro strap, on the lateral side of the shank and proximally to the knee, at approximately one third of the distance between the knee and the ankle joints. The employed sensor [21, 22] (Fig. 1) measures 50 × 90 × 10 mm, weighs 40 g (including battery), and was fitted with a high-performance low-power 32-bit microcontroller and a 9 DoF inertial sensor (incorporating an accelerometer, a gyroscope and a magnetometer), with range of 16 g and 2000 dps for the accelerometer and the gyroscope, respectively. The device was also equipped with a removable microSD card for on-board data storage, a Li-ion battery, a touch button for the operation of the device, and four LEDs for user feedback. The system can operate for more than 4 h, and the sampling rate was set at 246 Hz.

During their lab assessment, the operation and correct placement of the sensor was demonstrated to all participants. Subjects were asked to tighten the elastic band as much as possible without causing any discomfort in order for the device not to move from its initial placement during the recordings. Subsequently, videos of a physiotherapist performing five typical rehabilitation exercises for the lower limbs (i.e., supine knee extension, split squat, advanced clam, half squat and mountain climber) at the appropriate pace were shown to each participant in that order. We included isotonic exercises through the full RoM (i.e., knee extension supine and clam advanced), closed kinetic chain (CKC) exercises (i.e., spit squat and half squat) and cardiorespiratory training (e.g., mountain climber). Isotonic strengthening using the full RoM can be successfully used in the early stages of musculoskeletal rehabilitation, while CKC exercises are considered safe and effective when dealing with lower limb injuries and disorders, as they cause less shear forces to the joints and help improve the overall functionality of the person [23–25]. Additionally, as per the newest guidelines on physical activity [26], cardiorespiratory endurance training should be included in physical therapy prescriptions of all ages and disorders. The chosen exercises were also considered in previous research on physical therapy programs that focused, for example, in managing lower back pain [27], improving hamstring flexibility [28], and managing patellofemoral pain and ACL sprains [29]. The videos were also accompanied by verbal instructions on the correct execution of each exercise, and the recited text is included in Appendix 1 of this publication.

Consequently, participants were asked to perform six to eight repetitions of each exercise in a similar manner and speed to those demonstrated in the video recordings, with the inertial sensors collecting lower limb kinematics as part of the lab data collection session. No other instructions were given. Following the execution of the physiotherapy program in the laboratory, the sensor’s recordings were extracted, and each participant was given an identical sensor for at-home data capture. Additionally, detailed instructions with photographic material on the correct placement and operation of the device were provided to each participant, along with information on the correct execution of each exercise (e.g., Appendices 1 and 2). For the proper execution of each exercise, participants were asked to refer to their instructions as frequently as possible and follow the program for a week (daily, if possible), while also indicating in the provided diary, the days that the exercises were carried out and when they referred to the instructions. On the last day of this week-long program, they were also requested to capture their movements with the sensor while at home, and return the device on the following days. Recorded data are publicly available at Figshare [30], and further details on the format of the files are included in Appendix 3: Data availability.

### Data processing

The acceleration signal of the axis in which the movement predominantly occurs (e.g., the longitudinal axis in supine knee extension or the frontal axis in advanced clam) was used for the segmentation of the trial and the extraction of the trial’s section that corresponds to the recorded repetitions of each exercise. Subsequently, the number of movement cycles and the average cycle duration in seconds, for each exercise, were calculated by the number and periodicity of the peaks in the recorded signal. Then, as detailed in the following subsections, the signals from the wearable inertial sensors were post-processed to extract established features that have been previously used in literature to successfully characterise the smoothness, intensity and consistency of movements as captured with inertial wearable sensors [31–36]. Data processing was performed in Matlab (R2018a, MathWorks) in a fully automated fashion, and thus ensuring the unbiased treatment and comparison of the recordings.

### Log dimensionless jerk (LDLJ)—movement smoothness

The log dimensionless jerk, as calculated directly from the IMU’s acceleration signals, was used to obtain an index characterising the smoothness and hesitation in the executed movements. Since the LDLJ metric is very sensitive to measurement noise [37], the raw signals were filtered with a low-pass, second-order, zero-phase shift Butterworth filter with a cut-off frequency of 6 Hz. For a movement to be performed smoothly, the trajectory of the end-effector (e.g., hand or foot) that occurs between t_1_ and t_2_, should minimize the integrated squared jerk cost: ${\int }_{{t}_{1}}^{{t}_{2}}{\dddot{x}\left(t\right)}^{2}+{\dddot{y}\left(t\right)}^{2}+{\dddot{z}\left(t\right)}^{2}dt$, where $\dddot{x}\left(t\right)$, $\dddot{y}\left(t\right)$ and $\dddot{z}\left(t\right)$ are the derivatives of the sensor’s triaxial acceleration with respect to time. Previous research has demonstrated the significance of dimensionless jerk-based measures that are independent of movement duration and properly quantify deviations from smooth, coordinated movements [38]. Since the dimension of the integrated squared jerk cost is in squared length divided by the fifth power of time (i.e., $\frac{{length}^{2}}{{time}^{5}}$), the measure is multiplied by an appropriate factor (here, $\frac{t2-t1}{{a}_{peak}^{2}}$ in $\frac{{s}^{5}}{{m}^{2}}$) in order to obtain a dimensionless smoothness measure, while the natural logarithm improves the sensitivity and responsiveness of the metric. Thus, and as previously described by Melendez-Calderon and Shirota [39], the LDLJ metric is defined as:

1 $$LDLJ=-\mathrm{ln}\left(\frac{t2-t1}{{a}_{peak}^{2}}{\int }_{{t}_{1}}^{{t}_{2}}{\dddot{x}\left(t\right)}^{2}+{\dddot{y}\left(t\right)}^{2}+{\dddot{z}\left(t\right)}^{2}dt\right),$$

where ${a}_{peak}$ is equal to the magnitude of the peak total acceleration minus the mean total acceleration of the movement, and $t1$ and $t2$ represent the time at beginning and end of the recording, respectively. The LDLJ metric was previously reported to return values approximately from − 3 to − 10 in upper-limb movements of stroke survivors [39], where a number closer to zero corresponds to smoother movements.

### Euclidean norm of the acceleration—movement intensity (MI)

Movement intensity was defined as the Euclidean norm of the linear triaxial acceleration ($\ddot{x}\left(t\right)$, $\ddot{y}\left(t\right)$ and $\ddot{z}(t)$) of the wearable sensor, as measured in *g*:

2 $$MI(t)=\frac{\sqrt{{\ddot{x}(t)}^{2}+{\ddot{y}(t)}^{2}+{\ddot{z}(t)}^{2}}}{g},$$

The mean value of the movement intensity ($\overline{MI}$) and its variation ($MIV$) were previously used to quantify the intensity of exercises in clinical applications using wearable inertial sensors [33, 35]. The $\overline{MI}$ and $MIV$ metrics belong to a family of accelerometer processing methods (such as the ENMO, the Euclidian norm of the acceleration minus one [40]) with multiple applications in clinical settings [41, 42], that aim to quantify the intensity of physical activity and have been shown to have a strong relationship with energy expenditure [43]. Such metrics do not require any filtering of noise [42], and they can be used along with cut-off values to differentiate mild from moderate and vigorous physical activity [41]. Additionally, both $\overline{MI}$ and $MIV$ are independent of the sensor’s orientation and they take values close to one and zero g, respectively, in recordings of periodic movements without speed fluctuations and intensity bursts.

### Range of angular velocity (RAV)—velocity magnitude

As a measure of the velocity magnitude in rotational movements [33, 36], the range of the angular velocity (RAV, in rad/s) was computed by subtracting the minimum from the maximum values of the Euclidian norm of the raw angular velocity within each repetition, and then averaged over all repetitions of each recorded exercise.

### Kinetic value—work done

An indicator of the work done during each exercise (in m^2^/s^2^ or J/kg) was calculated from the squared integral of the total raw resultant acceleration over time, from the beginning until the end of each exercise, and divided by two [36]. This feature is an estimate of the work done during an activity and is based upon the principle that work equals to the change in the kinetic energy of the entire body. However, the derived value multiplied by body mass, is not directly proportional to the caloric expenditure during a task, due to the employment of the kinematics of the shank instead of the body’s centre of mass [44].

### Autocorrelation—movement regularity

Autocorrelation has been previously used to measure movement regularity (i.e., the similarity between neighbouring cycles of a signal) in waveforms with periodic patterns, such as the acceleration obtained from wearable sensors during gait [32, 34]. Initially, the N-sample long acceleration recording *a*_*i*_ (*i* = 1, 2, …, *N*) in which the movement primarily takes place (in this example, the transverse axis in a split squat) is repetitively compared with a delayed by *m* samples copy of itself with the following equation:

3 $$A= \frac{1}{N} \sum_{i=1}^{N-m}{a}_{i}{a}_{i+m},$$

**Fig. 2:**
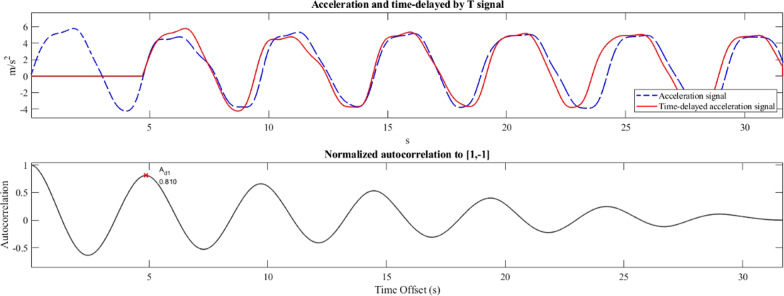
Autocorrelation example in a split squat exercise: transverse acceleration recording in blue and time-delayed by a period acceleration signal in red (top), and the autocorrelation plot (bottom) with the autocorrelation coefficient of the first (A_d1_) dominant period

For example, in Fig. 2 (top), the recorded transverse acceleration waveform during a split squat (in blue) is compared with an identical version of the signal which is time-delayed by a number of samples *m* that correspond to one movement cycle (in red), returning an autocorrelation coefficient equal to A_d1_ = 0.81. The sequency of the autocorrelation coefficients for every phase-shift *m* from 0 to N − 1, returns the autocorrelation function (Fig. 2, bottom). It should also be noted that when the phase-shift is equal to zero, the signal is compared with an exact copy of itself, and thus the amplitude of the autocorrelation takes its maximum value, which is also used to normalise the autocorrelation coefficients to a maximum of 1 (Fig. 2, bottom, vertical axis).

In an (almost) periodic waveform, when the phase shift *m* is equal to the periodicity of the signal, the two versions of the signal will be aligned (as in Fig. 2, top) and a peak will be found in the autocorrelation function. In this regard, the first peak (A_d1_) expresses the similarity between neighbouring exercise repetitions and it was used in this work to quantify the regularity in the execution of the physiotherapy exercises. Since the periodicity in the sensor’s acceleration signal is largely due to the change of the sensor’s orientation in respect to the direction of the gravity, a higher A_d1_ value closer to one, indicates that the subject performed each repetition consistently, without evident fluctuations in their movement speed, and with the same range of motion. Finally, since the autocorrelation process is sensitive to signal length (and recordings may have had dissimilar number of movement cycles), the coefficients were calculated for the first five repetitions of each exercise.

### Dynamic time warping (DTW)—movement stability

**Fig. 3:**
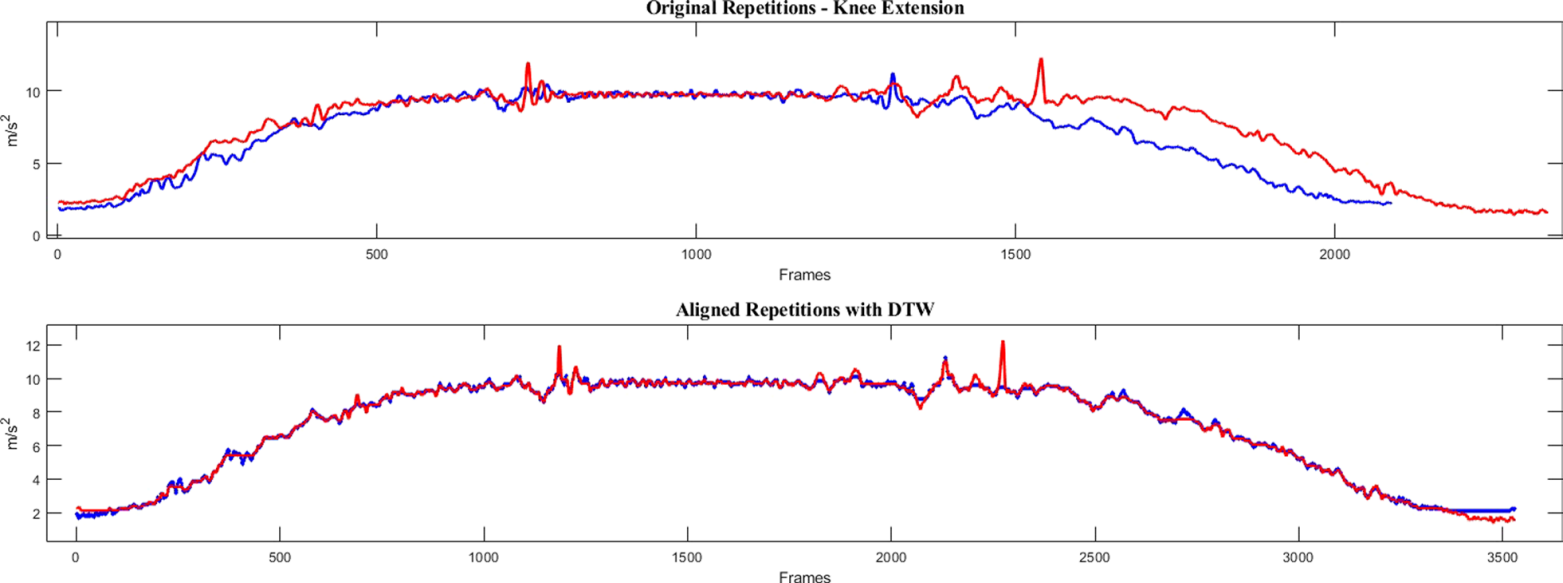
DTW example in a knee extension exercise: the longitudinal acceleration signals of two consecutive knee extension repetitions coming from the same recording (in red and blue) with different durations (top) are aligned with DTW (bottom)

Dynamic time warping (DTW), a technique widely used in audio signal processing and voice recognition, was used to compare the acceleration signals from successive repetitions of the same recording. The algorithm allows the comparison of two temporal signals with dissimilar lengths (e.g., Fig. 3, top), by finding the optimum non-linear alignment between them (Fig. 3, bottom) and returning a distance similarity measure that overlooks the periodicity of the signal (contrary to the autocorrelation coefficient). The DTW distance measure was calculated for every consecutive pair of repetitions normalised by length, and then averaged across all comparisons in the same recording. Analysis of acceleration signals with DTW has been previously used to compare the similarity of gait cycles and measure walking stability [35, 36, 45]. In the present work, a smaller DTW distance value may indicate a better ability to maintain control of joint movement throughout the execution of the exercises.

### Statistical analysis

The extracted featured between the two recordings (lab and home) were compared with mixed ANOVAs and with Tukey post-hoc tests. Assumptions of homogeneity of variances and sphericity were tested using Levene’s test and Mauchly's Test of Sphericity, respectively. Significance was set at P < 0.05.

## Results

According to diary entries, participants performed the exercise program almost daily, on an average of 6.3 ± 0.9 days within a week. Participants also reported consulting the provided instructive material on 2.1 ± 2.2 different days. No participant reported issues with the placement and operation of the sensors, or the recording of the exercises while at home.

During both data captures, participants executed all exercises faster (Table 1, cycle duration) compared to the demonstrated movements on the video footage by a physiotherapist (knee extension video demonstration: 12.5 s; split squat: 4.4 s; clam advanced: 8 s; half squat: 4.4 s; mountain climber: 2.2 s). On the occasions of the knee extension and mountain climber exercises (Table 1, in bold), the execution time was also further reduced when recording at home (*P* = 0.001 and *P* = 0.04, respectively).

**Table 1:** Measurements between assessments at the lab and home

Type of Exercise	Metrics																
	Cycle duration (s)			LDLJ		$\overline{\mathrm{MI}}$(g)		MIV (g)		RAV (rad/s)		Kinetic value (joule/kg)		Regularity		DTW (m/s^2^)	
	Lab	Home	Demo	Lab	Home	Lab	Home	Lab	Home	Lab	Home	Lab	Home	Lab	Home	Lab	Home
Knee Extension	**7.5 ± 1.5**	**5.8 ± 1.9**	12.5	− **8.2 ± .51**	− **7.9 ± .60**	.99 ± .01	.99 ± .01	.06 ± .02	.07 ± .02	.41 ± .10	.41 ± .12	**8.6 ± 13.5**	**3.3 ± 4.0**	.77 ± .05	.78 ± .05	53.0 ± 16.5	53.4 ± 19.0
Split Squat	3.7 ± .63	3.9 ± 1.2	4.4	− 7.9 ± .62	− 7.9 ± .73	1.0 ± .01	1.0 ± .01	.06 ± .03	.06 ± .02	.36 ± .09	.36 ± .09	1.9 ± 2.2	1.3 ± 1.5	.73 ± .10	.72 ± .09	97.1 ± 27.1	97.8 ± 40.0
Clam Advanced	4.0 ± 1.0	3.8 ± 1.7	8.0	− **7.7 ± .48**	− **7.5 ± .58**	1.0 ± .01	1.0 ± .01	.07 ± .02	.08 ± .04	.46 ± .11	.50 ± .16	7.3 ± 15.2	6.6 ± 14.0	.80 ± .07	.79 ± .07	83.0 ± 46.2	77.1 ± 36.0
Half Squat	3.5 ± .86	3.6 ± 1.5	4.4	− 7.8 ± .56	− 7.8 ± .56	1.0 ± .01	1.0 ± .01	.03 ± .01	.04 ± .02	.27 ± .08	.28 ± .13	2.2 ± 2.5	2.1 ± 3.2	.81 ± .04	.78 ± .07	62.7 ± 25.3	65.1 ± 25.0
Mountain Climber	**1.7 ± .27**	**1.5 ± .40**	2.2	− 8.5 ± .63	− 8.6 ± .68	1.6 ± .15	1.6 ± .22	.90 ± .28	.89 ± .38	2.4 ± .56	2.3 ± .74	1435 ± 959	1957 ± 1512	.38 ± .11	.38 ± .09	660.3 ± 150.7	713.0 ± 215.5

In bold: Significant main effect of time

The LDLJ metric displayed sensitivity for the characterisation of movement smoothness, with values ranging from − 8.6 to − 7.5, across all recordings. Our results confirm that the LDLJ metric is task dependant and exercises with more eminent alterations between accelerations and decelerations are less smooth. For example, the recorded mountain climber signals were significantly less smooth (lab: − 8.5 ± 0.63 and home: − 8.6 ± 0.68) than all other exercises performed both at home (*P* < 0.001), and at the lab (*P* < 0.007) with the exemption of the knee extension exercise (*P* = 0.35). Additionally, participants appeared to perform the knee extension (*P* = 0.006) and clam advanced (*P* = 0.026) exercises significantly smoother when at home.

The mean magnitude of the acceleration signal over the entire recording (Table 1, $\overline{\mathrm{MI}}$), was equal to 1 g for all the exercises that were executed at a constant speed and with controlled accelerations and decelerations at the beginning and end of each cycle (i.e., knee extension, split squat, clam advanced and half squat). Mountain climber recordings showed significantly higher movement intensity values compared to all other exercises (*P* < 0.001). The variability of the mean values of the acceleration magnitude (MIV) was also significantly higher in the mountain climber task (*P* < 0.001), indicating intense and multiple acceleration and deceleration phases.

The range of angular velocity (Table 1, RAV) metric was very consistent across all data captures in the lab and at-home, alike. The mean kinetic value of the knee extension recordings was significantly smaller at home, seemingly due to the significantly shorter durations of the exercise during the participants’ self-measurement. As expected, the kinetic values calculated from the mountain climber recordings were significantly higher compared to all other exercises (*P* < 0.001).

Regularity values were, on average, approximately equal to 0.38 for the mountain climber exercise, exhibiting the inherent difficulty of the task to be performed from one repetition to another in a concise and periodic fashion. On the other hand, the mean regularity values for all the other recordings were fairly high, with values ranging from 0.72 to 0.81. In support of the assumption that the DTW metric is associated with balance and dynamic control of the movement, the distance measures were the lowest for the less complex exercises that participants were lying flat on their back (knee extension) or with both feet stable on the ground (half squat), followed by the tasks that require some degree of movement control and coordination (split squat and clam advanced), while the values associated with the mountain climber recordings were significantly higher.

## Discussion

Previous works on tracking exercise performance have employed videotape recordings [8, 9] or live observation [10, 11] of exercise execution in order for trained assessors to review and score the participants’ movements. In contrast, the present work accounts for potential assessor and observation biases, by evaluating the subjects’ performance when at-home, with the use of wearable sensors and automated algorithms that can assess the participants’ ability to reproduce the studied movements. Our system showed that participants were very consistent in the execution of all exercises at-home as compared with their performance in the lab, with only a few exceptions in the cycle duration (Table 1, knee extensions and mountain climbers), movement smoothness (knee extensions and clam advances), and kinetic output (knee extensions).

The employed wearable system additionally showed that participants could not effectively set the pace for the execution of all exercises as compared to the demonstrated movements of the physiotherapist during their lab session (Table 1, cycle duration: demo). Since therapeutic exercises need to be executed consistently and with appropriate intensity to optimise therapy outcomes [46], a wearable system with real-time objective performance metrics and feedback (implemented either with virtual reality platforms, real-time visualizations in a mobile application, or haptics) would be essential for the proper execution of a therapy program without supervision. Additionally, considering that participants tend to be biased when self-reporting frequency of home activities [5, 47], a wearable system with date-time stamps such as the one presented in this study, could objectively measure the number of times patients attempted an exercise session at home.

Even though our results indicate that a single-sensor solution may suffice for the monitoring of physiotherapy programmes (as also demonstrated in [48]), a second sensor on the thigh could also be used for the calculation of the knee’s range of motion (RoM) and thus, help better assess how well people carry out lower-limb exercises when at-home. For example, the consistency in the magnitudes of the RAV values between laboratory and home measurements, along with the significant reduction in the cycle duration of the knee extension and mountain climber exercises when at-home, may indicate that participants performed the two exercises with a significantly smaller RoM when recording alone: the RAV metric indicates that participants performed the knee extension exercise at home as slowly as in the lab, but an early termination of a repetition without adequately flexing the knee in order to bring the foot close to the bottoms, may be the cause behind the smaller cycle durations and kinetic values. In this context, either a single IMU sensor in conjunction with machine learning algorithms [49, 50], or a two-sensor system could confirm that flexion/extension was the principal motion on both occasions, and also return the RoM of the movements.

Concerning the analysis of movement smoothness, a number of different factors should be taken under consideration when evaluating recordings from IMU data. The selected LDLJ measure is reported to be one of the most appropriate metrics for translational movements [39], but a low-pass filter is recommended to supress high frequency noise [37]. Since different filters will significantly affect smoothness estimates, the entire dataset should be treated uniformly. In recordings where rotational movements are more evident, the spectral arc-length measure (SPARC), along with the angular velocity signal from the gyroscope, may be used instead of the LDLJ [51]. Finally, when dealing with recordings of rhythmic movements (e.g., walking or multiple repetitions of an exercise) with significantly dissimilar number of movement cycles or movement speeds, it is advisable to segment each recording to distinct not overlapping movement components, and estimate the recording’s overall smoothness by calculating the weighted average smoothness of the individual components [37].

In summary, this feasibility study illustrated how the developed sensor and the employed metrics may be relevant in assessing exercise performance in healthcare and telemedicine. In a previous study with clinicians expressing their thoughts on the use of IMUs in their practice [52], measures assessing movement quality, such as those in the present work (e.g., LDLJ, regularity and DTW), were ranked among the ten main categories of variables that are useful to measure in a clinical setting. Additionally, the developed IMU-based wearable system features a range of desirable characteristics that makes it appropriate for clinical use [52, 53], since it is easy to use and wear, is comfortable, sturdy, lightweight and easy to generate results, while it has only one sensor and not too many control buttons, is cost-effective, it has increased battery life, and is accompanied by light indications when the device is working. Most importantly, the potential usefulness of this technology in clinical practice stems from its capability to monitor patient progress, facilitate engagement in care and telecare, establish a quantitative profile characterising each exercise session, and gather quantitative data related to physical exercises that are difficult to assess due to their complexity. On that matter, this study is additionally accompanied by preliminary normative data [30] that are scarce (another example is [54]) and can be of particular value to researchers working on wearables in telemedicine.

A limitation of the present work springs from the recruitment of only young, healthy adults for the evaluation of a week-long therapy program. Patients that are following an exercise programme at-home as part of their physical therapy, may perform differently over time, and as their treatment plan elevates pain, swelling and discomfort. Thus, uninjured subjects were preferred in this study in order to control for such factors (e.g., muscle weakness, pain, limited endurance, pain flare-ups) that may affect movement. Finally, since exercise demonstration was recent for the participants with only a week between the initial video demonstration and performing the exercises at-home, physical programs with a longer time duration should be investigated for the evaluation of metrics assessing exercise performance.

## Conclusions

This work presents a novel approach of using a single wearable sensor and IMU derived features and metrics for the quantification of exercise performance. All the considered metrics appear to be appropriate and exercise-sensitive to evaluate home-based programmes in an unsupervised manner. Our analyses suggest that participants did not execute the prescribed movements at the same pace as the physio’s demonstrations in the video footages, indicating the potential benefit of a cost-effective and patient-centered wearable system with user real-time feedback for the consistent and appropriate execution of home-based therapy programmes. The next steps to complete this feasibility study include the use of the developed wearables and metrics by physical therapists and during home programmes for prolonged periods of time, while also gathering qualitative feedback from patients and physicians to determine the needs of the stakeholders and the gaps in the developed wearable system.

## Data Availability

The data that support the findings of this study are openly available in figshare at http://doi.org/10.6084/m9.figshare.13483599, [30].

## Appendix 1: Instructions

*Knee Extension Supine*: Begin by lying on your back with your dominant knee bent (the one with the sensor) and the foot resting on the floor, while your other leg is straight and relaxed. Slide your heel away from your bottom to straighten your dominant leg. Go as far as feels comfortable and try to straighten your leg at the bottom by “pushing” you knee down towards the ground. Return to the start position. Repeat 5–6 times.

*Split Squat*: Start in a split leg position, with one leg forward (the one with the sensor) and one leg back. Make sure your front knee is directly over the second ray of your foot and in good alignment. Your knee should never drop inwards. Contract your VMO, the inside of the front of your thigh muscle group, and your buttock muscles of your front leg and slowly drop your back knee towards the ground. Your front knee stays at 90 degrees, but does not go forwards of that point. Return to the start position. Repeat 5–6 times.

*Clam Advanced*: Lie on your side, with your dominant leg on top and both knees bent. Squeeze your deep abdominal muscles by drawing the belly button inwards. Keeping your feet together, lift the feet 3–4 inches above the floor. Open your knees, like a clam, hold, and return to the start position. Repeat 5–6 times.

*Half Squat*: Open your legs slightly wider than shoulder width, and bend your knees to a half squat position. Make sure you keep the middle of your knee-cap in line with the middle toes of your foot. Repeat 5–6 times.

*Mountain Climber*: Put both hands and knees on the floor. Place your right knee near your right arm and extend your left leg behind you. In one smooth motion, take it back out and then bring your left knee in towards your left arm, while keeping your arms in the same position. Repeat 10–12 times.

## Appendix 3: Data availability

Recordings, from both sessions, are publicly available at Figshare in Excel spreadsheets (http://doi.org/10.6084/m9.figshare.13483599) [27]. The name of each file is a concatenation of the subject’s identifier (1–30), session number (1: lab or 2: home), type of instructions (video, written or illustrations) and type of exercise (e.g., Subj_1_Visit_1_Video_KneeExtension). Each file contains the sensor’s time stamp in seconds, and triaxial acceleration (m/s^2^) and gyroscopic (rad/s) data. Sensor placement was such that the x, y and z axes of the sensor were aligned with the longitudinal, transverse and frontal axes of the shank.
